# Anticipated Impact of Exoskeleton Use on Older Workers’ Occupational Self-Efficacy: A Survey Study

**DOI:** 10.1093/geroni/igaf122.2177

**Published:** 2025-12-31

**Authors:** HeeSun Choi, Sahar Khanpour, Yves Valentin, Jessica Alquist, Jonathan Singer

**Affiliations:** Texas Tech University, Lubbock, Texas, United States; Texas Tech University, Lubbock, Texas, United States; Texas Tech University, Lubbock, Texas, United States; Texas Tech University, Lubbock, Texas, United States; Texas Tech University, Lubbock, Texas, United States

## Abstract

Exoskeletons are wearable assistive technology that can reduce users’ physical strain and enhance performance, having significant health and safety implications for older manual labor workers with age-related declines. However, their psychosocial impact remains understudied; exoskeleton use may enhance occupational self-efficacy (OSE) – beliefs in one’s ability to perform work tasks, by helping to regain functional capabilities and successfully meeting day-to-day work demands. This survey study explored whether older manual labor workers anticipated changes in OSE with exoskeleton use, using a hypothetical scenario. Additionally, we examined whether perceived embodiment (i.e., perceiving exoskeletons as an extension of their body vs. an external tool) influenced the impact of exoskeletons on OSE. Forty-four participants aged 55 + (M = 62.59, SD = 6.24) rated their baseline OSE, followed by watching an exoskeleton introduction video. They were then asked to indicate their anticipated OSE, assuming their workplace implemented exoskeletons and they had been using them for 12 months. A repeated measures ANCOVA revealed a significant main effect, with post-video OSE ratings (M = 25.59, SD = 6.55) lower than baseline (M = 30.32, SD = 3.39), F(1,42) = 17.84, p < .001, ηp² = .30. Additionally, perceived embodiment moderated this effect, F(1,42) = 10.18, p = .003, ηp² = .20. Workers with low embodiment anticipated reduced OSE, while those with high embodiment did not. Findings suggest exoskeletons may negatively impact older workers’ OSE, particularly if perceived as external tools. Future research should explore this effect in larger samples and assess real-world changes following hands-on experience.

